# The role of TRPV1 in chronic prostatitis: a review

**DOI:** 10.3389/fphar.2024.1459683

**Published:** 2024-09-19

**Authors:** Zhipeng Jiang, Wen Luo, Zongmin Long, Jie Chen

**Affiliations:** ^1^ Third Affiliated Hospital of Zunyi Medical University (First People’s Hospital of Zunyi), Zunyi, China; ^2^ Kweichow Moutai Hospital, Zunyi, China

**Keywords:** TRPV1 channel, prostatitis, pain, lower urinary tract symptoms, therapeutic target

## Abstract

Chronic prostatitis is a prevalent male urinary system disorder characterized by pelvic discomfort or pain, bladder dysfunction, sexual dysfunction, and infertility. Pain and lower urinary tract symptoms (LUTS) are the most common symptoms, significantly impacting patients’ quality of life and driving them to seek medical attention. Transient receptor potential vanilloid subtype 1 (TRPV1) is a non-selective calcium ion-dependent cation channel in the TRPV channel family that is widely distributed in neural tissue and plays a role in signal transmission. In this review, we provide a comprehensive overview of the current understanding of the role of TRPV1 in chronic prostatitis. The discussion focuses on the connection between TRPV1 and prostatitis pain and LUTS, and highlights the potential for targeting this channel in the development of novel treatment strategies.

## 1 Introduction

Prostatitis is characterized by an inflammatory response triggered by microbial pathogens infecting the prostate or by certain non-infectious factors. The incidence rate of prostatitis is approximately 4.5%–9%, with a recurrence rate of around 50% in elderly patients (Polackwich and Shoskes, 2016). In 1995, the National Institute of Health (NIH) classified prostatitis into four categories. Among these categories, type III prostatitis, known as chronic prostatitis/chronic pelvic pain syndrome (CP/CPPS), accounts for more than 90% of cases. Its primary clinical manifestations include discomfort or pain in the pelvic area, abnormal urinary function, sexual dysfunction, and infertility. Pain and lower urinary tract symptoms (LUTS) are the most prevalent and impactful symptoms, significantly affecting patients’ quality of life and being the primary reasons for patient visits (Pena et al., 2021). At present, the primary factors contributing to chronic prostatitis are latent infection, autoimmunity, neuroendocrine factors, sex hormone disorders, pelvic floor muscle dysfunction, peripheral and central sensitization, neuroplasticity, and psychosocial disorders (Lai et al., 2015; Bharucha and Lee, 2016). Nevertheless, the precise mechanism underlying chronic prostatitis remains elusive. Cross-organ sensitization has been a popular theory for the past decade, whereby irritation from an affected pelvic organ can be transmitted to nearby healthy organs, causing healthy organ dysfunction. A study found that sensory nerves from the prostate and bladder converge in the lumbosacral dorsal root ganglion (DRG) (Lee et al., 2016; Schwartz et al., 2016; Funahashi et al., 2019). Research by Aydogdu et al. (2021) and Funahashi et al. (2019) suggested that LUTS in prostatitis may be associated with sensitization of the prostate-bladder organ. Clinically, the main treatments for prostatitis include pharmaceutical treatments (antibiotics, alpha-blockers, anti-inflammatory/immunomodulatory drugs, etc.) and non-drug treatments (Franco et al., 2019; Pena et al., 2021). However, in certain cases, although the inflammation of the prostate tissue resolves after treatment, the symptoms of prostate inflammation persist, highlighting the need for the development of new treatment approaches.

In recent years, some studies have indicated that the transient receptor potential vanilloid subtype 1 (TRPV1) channel is significant in the pathogenesis and progression of prostatitis. TRPV1 is a member of the TRPV channel family, responding to various stimuli such as mechanical, thermal, and chemical cues from both intracellular and extracellular environments (Aghazadeh Tabrizi et al., 2017). This channel is found in both neural and non-neural tissues (Bevan et al., 2014). Activation of TRPV1 by different substances can result in nociceptive or neuropathic pain responses. For instance, certain pro-inflammatory molecules can trigger inflammatory pain by acting on TRPV1, leading to increased expression of this channel in neurons and intensifying the pain response (Aghazadeh Tabrizi et al., 2017). Previous research has identified the expression of TRPV1 in both prostate tissue and prostate innervating nerves (Dinis et al., 2005; Zhang et al., 2019; Vanneste et al., 2021). Following prostate inflammation, an upregulation of TRPV1 expression was noted in bladder tissue, prostate tissue, and prostate-bladder co-innervated neurons (Zhang et al., 2019). Therefore, further elucidating the role of TRPV1 in prostatitis can enhance our understanding of the underlying mechanisms of this condition.

This review provides an overview of the TRPV1 channel structure and explores its expression in prostate tissue and innervation, aiming to understand the role of TRPV1 in pain and LUTS associated with prostatitis. The findings have important implications for future research on the pathogenesis and potential treatment of prostatitis.

## 2 Overview of TRPV1 channels

**FIGURE 1:**
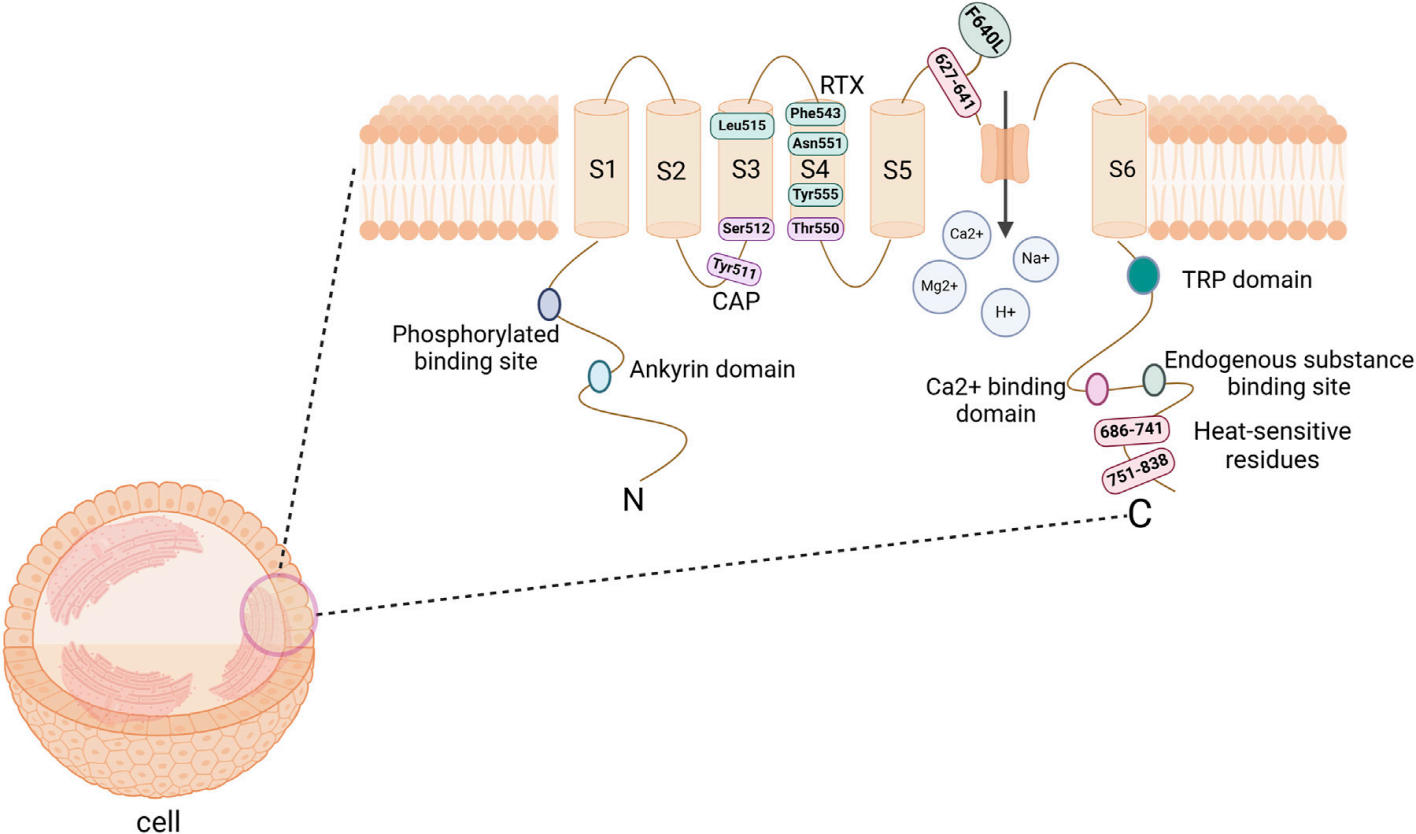
The structure of TRPV1 channel protein. It consists of a tetramer, with each subunit comprising 6 transmembrane domains (S1-S6) on the cell membrane, along with the N-terminus and C-terminus within the cell. The hydrophobic group formed by the transmembrane domains forms a pore that allows cations to pass through. The N-terminal serves as a site for phosphorylation and ankyrin binding, while the C-terminal acts as a binding site for endogenous substances and calcium ions. CAP binding sites are Tyr511, Ser512, and Thr550; RTX binding sites are Leu515, Phe543, Asn551, and Tyr555; Heat-sensitive binding sites are 686–741, 751–838, and F640L. Abbreviations: TRPV1, transient receptor potential vanilloid subtype 1; CAP, Capsaicin; RTX, Resiniferatoxin. Image was created with BioRender.com.

TRPV1 is a non-selective cation channel that is located on the plasma membrane of cells, enabling the passage of cations such as H^+^, Na^+^, Ca2^+^, and Mg2^+^ (Caterina et al., 1997). The TRPV1 protein exhibits a homotetrameric structure, with each subunit consisting of three main parts (Maximiano et al., 2024): six transmembrane domains (S1-S6) located on the cell membrane, which form a hydrophobic pore; an intracellular N-terminus that includes six ankyrin repeat domains and multiple phosphorylation sites; and a C-terminus that contains a TRP domain, endogenous substance binding sites, and multiple calmodulin-binding domains (Figure 1).

TRPV1 channels are an integration of multiple noxious stimulation molecules and can be activated by various thermal, physical or chemical stimuli (Maximiano et al., 2024). In an environment with a neutral pH, the channel is triggered by harmful heat (>43°C). However, in settings where the pH levels are decreased (like in cases of inflammation or ischemia), the receptor may be activated even at room temperature (Caterina et al., 1997; Frias and Merighi, 2016); In particular, the temperature-sensitive response of TRPV1 is associated with the C-terminal domain, particularly the representative domain sequences 686–741 and 751–838, as well as certain residues in the pore region, such as F640L within the 624–641 sequence (Bevan et al., 2014). In addition, endogenous substances such as cannabinoids, Oleoylethanolamine (OEA), palmitamide ethanol (PEA), bradykinin, and growth factors can modulate the expression of this receptor; Exogenous substances such as capsaicin (CAP), resiniferatoxin (RTX), ethanol, and vanillin have been discovered to stimulate the receptor (Frias and Merighi, 2016; Maximiano et al., 2024). Research has pinpointed the importance of TM3 and TM4 in this channel for interacting with the agonists CAP and RTX (Bevan et al., 2014). Notably, the key binding sites for CAP include Tyr511, Ser512, and Thr550, while the significant binding sites for RTX are Leu515, Phe543, Asn551, and Tyr555 (Andrei et al., 2023). Although the exact binding sites for other agonists remain unclear, there is a hypothesis suggesting potential overlap with the known binding sites (Bevan et al., 2014).

TRPV1 acts as an ion channel that allows high permeability to Ca2^+^ (Caterina et al., 1997). Activation of this channel leads to the influx of extracellular Ca2+ into the cell, which in turn triggers the release of intracellular Ca2+ stores. This process ultimately results in an increase in intracellular calcium ions, causing cell depolarization and the generation of an action potential. Subsequently, a cascade of intracellular signals is initiated, leading to the release of neurotransmitters such as calcitonin gene peptide, somatostatin, and SP substances. These events play a crucial role in triggering multiple signaling cascades (Caterina et al., 1997; Frias and Merighi, 2016; Satheesh et al., 2016; Maximiano et al., 2024).

TRPV1 is widely expressed in neural and non-neural tissues. The expression of this protein is predominantly found in small to medium-sized neurons within the DRG, trigeminal ganglia, and vagal ganglia. These neurons give rise to unmyelinated C-type sensory nerve fibers or myelinated Aδ-type sensory nerve fibers that innervate various organs and tissues (Caterina et al., 1997; Ho et al., 2012; Bevan et al., 2014; Bujak et al., 2019). Nonneuronal expression of TRPV1 can be found in arteriolar smooth muscle (Phan et al., 2020). Relevant evidence suggests that TRPV1 channels play a role in multiple physiological functions (Szallasi and Blumberg, 1999). Roman et al. (2020) utilized TRPV1 antagonists to mitigate hyperalgesia induced by prostatitis. However, the absence of pelvic allodynia in TRPV1 knockout mice suggests a direct correlation between TRPV1 channels and pain. Therefore, we can infer that The abnormal activation or overactivation of TRPV1 channels is likely associated with the development and pathophysiological progression of the disease. In certain instances, excessive activation of TRPV1 channels can result in heightened pain sensitivity and inflammatory reactions.

## 3 Expression and function of TRPV1 channel in normal prostate, bladder and DRG

In human prostate tissue, TRPV1- immunoreactive (-IR) fibers are present in the prostate urethral mucosa, seminal fluid, ejaculatory ducts, and periurethral prostate acini. Conversely, TRPV1-IR fibers are not detected in the transitional zone and peripheral zone of the gland (Dinis et al., 2005). In animal models of experimental autoimmune prostatitis, TRPV1 expression was found to be upregulated not only in the prostate but also in the L5-S1 DRG and bladder tissue (Zhang et al., 2019).

Under a microscope, bladder tissue is divided into mucosal, muscular, and adventitial layers. Within the bladder mucosa, the majority of TRPV1-IR fibers are positioned near the basal cells of the bladder transitional epithelium. In the bladder muscle layer, TRPV1-IR fibers exhibit a close association with smooth muscle cells (Lazzeri et al., 2004; Lazzeri et al., 2009). Early pharmacological studies have demonstrated that capsaicin-sensitive afferent fibers (C-fibers) are crucial in the micturition reflex. These fibers have a dual role, acting as sensory afferents in regulating the micturition threshold and vesicourethral pain perception, as well as efferents in controlling nerve excitability, contractility of smooth muscle, and extravasation of plasma proteins (Lazzeri et al., 2009).

In the DRG, TRPV1 is primarily found on the plasma membrane and endoplasmic reticulum of neurons. Activation of TRPV1 leads to the release of Ca2^+^ from the endoplasmic reticulum, influencing synaptic and presynaptic growth. This process is crucial for signal and synaptic transmission (Wong et al., 2014). These sensory neurons transmit sensory information to various internal organs including the prostate, periurethral area, ejaculatory ducts, skin, and muscles of the genitourinary tract (Light et al., 2008), resulting in a range of biological responses. TRPV1 sensory nerves located in the prostate and perineal skin contribute to heightened pain sensitivity in individuals with chronic prostatitis/chronic pelvic pain syndrome (Lazzeri et al., 2009). Preclinical studies have shown that DRG is the key to this phenomenon (Grundy and Brierley, 2018). Nerve damage, neuropathy, and inflammation in the pelvic organs may cause an upregulation of DRG neurons. This heightened activity can persist as a result of neuroplasticity at the peripheral nerve terminals, DRG neurons, and spinal cord (Grundy and Brierley, 2018; He et al., 2023).

Therefore, the presence of numerous TRPV1 sensory nerves in the prostate and bladder suggests a potential new approach for treating pain and LUTS in patients with chronic prostatitis.

## 4 Relationship between TRPV1 channels and CP/CPPS

### 4.1 Association between TRPV1 and autonomic nervous function of CP/CPPS

The autonomic nervous system plays a significant role in the development of chronic pelvic pain syndromes (CP/CPPS). This system encompasses both the peripheral autonomic nervous system, which includes sensory, sympathetic, and parasympathetic nerves, and the central nervous system, comprising the spinal cord and brain (He et al., 2023). During the development of CP/CPPS, alterations in the autonomic nervous system are accompanied by changes in associated substances, receptors, and ion channels. These include substance P (SP), calcitonin gene-related peptide (CGRP), nerve growth factor (NGF), prostaglandin E2 (PGE2), TRPV1 channel, tropomyosin receptor kinase A (TrkA), and protease-activated receptor 2 (PAR2), among others. In this review, we will focus exclusively on the alterations of TRPV1 channels within the autonomic nervous system in the context of CP/CPPS.

The sensory nervous system plays a crucial role in mediating neurogenic inflammation and peripheral sensitization in CP/CPPS. When nociceptive receptors in prostate tissue are repeatedly stimulated by noxious stimuli, C-fibers transmit nociceptive signals to the spinal cord and central nervous system via the DRG. During this process, these sensory nerve fibers activate and release neurogenic peptides, such as SP and CGRP, which mediate neurogenic inflammation and peripheral sensitization (Birder and Kullmann, 2018; He et al., 2023). The upregulation of neurotransmitters and channel receptors within the DRG can activate and sensitize sensory nerves, while also regulating organ cross-sensitization due to the bipartite afferent properties of the DRG (He et al., 2023). TRPV1 plays a crucial role in pain perception within the sensory nervous system (Shiers et al., 2020). The activation of this channel results in an increase in intracellular calcium ion concentration and membrane depolarization, which facilitates the transmission of pain signals as electrical impulses to peripheral nerve fibers, spinal nerves, and cranial nerves (Moore et al., 2017). Consequently, the intrinsic properties of the TRPV1 channel contribute to a lowered pain threshold within the sensory system associated with prostatitis, thereby intensifying the perception of pain. This channel plays a crucial role in mediating the persistent pain experienced in prostatitis.

Sympathetic and parasympathetic nerves originate from the hypogastric plexus. During the onset and progression of prostatitis, alterations in these nerve pathways influence the persistence of pelvic pain and contribute to cross-sensitization between organs (He et al., 2023). The destruction of sympathetic nerves or the release of catecholamines may contribute to the development of chronic prostatitis (Hu et al., 2022; He et al., 2023). Additionally, the imbalance between sympathetic and parasympathetic nerves resulting from chronic prostatitis is a contributing factor to organ cross-sensitization. This sensitization affects various systems, including the cardiovascular, reproductive, and urinary systems. In this review, we will focus exclusively on bladder dysfunction associated with prostatitis. Under normal circumstances, the innervation of the bladder comprises both afferent and efferent nerves. Afferent nerves gather peripheral sensory information and relay it to the spinal cord, while efferent nerves primarily consist of sympathetic and parasympathetic fibers. The interplay between these two types of nerves collaboratively regulates the activity of urination. When the bladder is full, sympathetic nerves release norepinephrine to facilitate smooth muscle relaxation. Conversely, during bladder emptying, parasympathetic nerves release acetylcholine, which induces contraction of the bladder (Yoshimura et al., 2014; Miyazato et al., 2017; Birder and Kullmann, 2018). The sensitization of bladder afferent nerves and the associated material changes induced by CP/CPPS will be discussed in the subsequent chapters. Concerning the efferent nerve activity of the bladder, Lepiarczyk et al. (2017) demonstrated that the number of cholinergic and norepinephrine nerve fibers decreased following the administration of TRPV1 agonists in the bladder. The intraspinal administration of TRPV1 agonists has been shown to decrease sympathetic activity (Wu et al., 2020). These studies have demonstrated a significant relationship between TRPV1 and both sympathetic and parasympathetic nerves. Consequently, during the progression of chronic prostatitis, alterations in TRPV1 may indirectly or directly influence the sympathetic and parasympathetic nervous systems of the prostate and bladder, thereby exacerbating the development and functional changes associated with chronic prostatitis.

The central nervous system, comprising the spinal cord and brain, integrates and processes nociceptive signals. Central sensitization can reduce the pain threshold and heighten the perception of pelvic pain in chronic prostatitis, thereby contributing to the onset and progression of persistent pelvic pain (Birder and Kullmann, 2018; He et al., 2023). Furthermore, the spinal cord segment at the lesion can influence changes in related substances within the adjacent healthy spinal cord segment, indicating that spinal cord sensitization also plays a role in pain sensitization (Javed et al., 2020). In the central nervous system, TRPV1 is predominantly expressed in layers I and II of the dorsal horn of the spinal cord, where it plays a crucial role in regulating the synaptic transmission of nociceptive signals originating from the periphery (Jeffry et al., 2009; Spicarova and Palecek, 2009). Following intraspinal administration of TRPV1 agonists, the connection between TRPV1-expressing neurons and the spinal cord may be permanently disrupted (Iadarola and Gonnella, 2013), resulting in prolonged analgesia (Jeffry et al., 2009).

### 4.2 TRPV1 and pain caused by CP/CPPS

Activation of TRPV1 in humans triggers nociceptive neurons in the lower urinary tract, causing pain and burning sensations (Maggi et al., 1989). Interestingly, this is the predominant description of pain during urination or ejaculation in patients with CP/CPPS. Dinis et al. (2005) demonstrated that urination, ejaculation, and perineal pain in patients with CP/CPPS are directly related to activation of TRPV1-IR fibers. TRPV1 receptors are thought to be critical for pain perception in prostatitis patients (He et al., 2023). Animal experimental studies have demonstrated that the expression of the nociceptive substance TRPV1 in sensory neurons innervating the prostate increases during prostatic inflammation. Furthermore, the loss of TRPV1 channels has been shown to disrupt the progression of pelvic abnormal pain (Schwartz et al., 2015; Roman et al., 2020).

Prostatic pain exhibits characteristics of visceral organ pain, with pain sensation being transmitted through C-fibers nerves (Lee et al., 2001). The majority of TRPV1 fibers co-express the neuropeptides SP and CGRP, which are commonly associated with pain perception (He et al., 2023; Marek-Jozefowicz et al., 2023). Therefore, inflammatory sensitization of TRPV1 channels on C-fibers nerves plays a crucial role in the development of persistent pain (Malek et al., 2015). First, after prostate inflammation, various cells produce pro-inflammatory factors (TNF-α, IL-8, IFN-γ), anti-inflammatory factors (IL-4, IL-6, IL-10), and regulatory factors (CD4^+^). Imbalances in these factors contribute to the progression of pain (Wang et al., 2023), with mast cells playing a central role among these cells (Zheng et al., 2017; Li et al., 2023). Following mast cell activation, the cell undergoes degranulation and releases pain-inducing molecules such as NGF and trypsin-β (Done et al., 2012; Roman et al., 2014). NGF, in particular, enhances the expression and transportation of ion channels (e.g., TRPV1) to increase the excitability of nociceptive neurons (Zhang et al., 2005; Stein et al., 2006). Moreover, NGF is involved in regulating the expression of pro-inflammatory molecules like SP and CGRP on nociceptive neurons (Donnerer et al., 1992). Secondly, inflammation triggers the activation of C-fibers nerves, leading to the release of pain transmitters in central terminals. This process also initiates multiple reverse impulses in sensory afferent nerve fibers through local axon reflexes and spinal dorsal root reflexes. Additionally, it involves the release of various neuroactive substances, such as SP and CGRP, resulting in neurogenic inflammation characterized by local vasodilation, congestion, and plasma protein extravasation. Furthermore, some DRG neurons have axon branches connected to neighboring pelvic visceral structures, enabling retrograde propagation of action potentials that may cause neurogenic inflammation in these adjacent pelvic visceral structures, potentially contributing to persistent and referred pain beyond the prostate region (Foreman, 1987; He et al., 2023). And neurogenic substances released from nerve endings can stimulate mast cells to produce additional neuropeptides like NGF, cytokines, and chemokines (Theoharides et al., 2012; Birder and Kullmann, 2018; Lauritano et al., 2023). This process can exacerbate pain sensitization, thereby contributing to the development of a vicious cycle.

**FIGURE 2:**
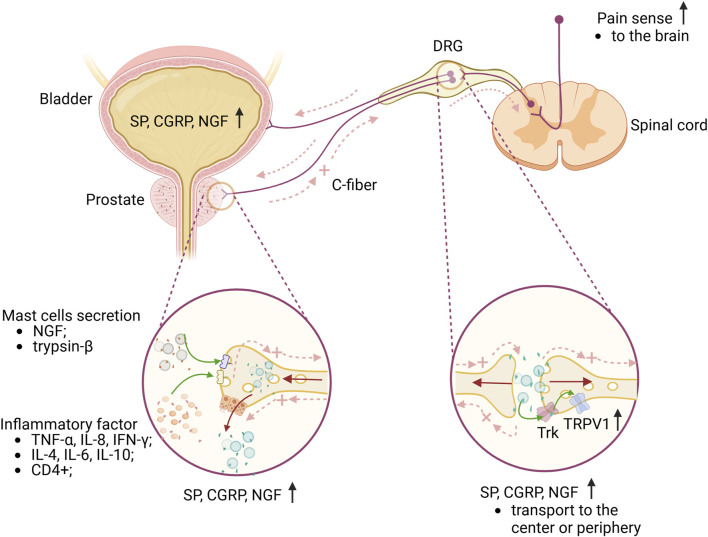
Briefly explain the mechanism of CP/CPPS pain and lower urinary tract. The accumulation of inflammatory factors in the prostate, along with the activation and degranulation of mast cells, results in the sensitization of C-fibers nerve endings in the prostate. This sensitization causes the DRG neurons, which innervate both the prostate and bladder, to express elevated levels of SP, CGRP, and NGF. The release of these neuropeptides into the central nervous system contributes to pain sensitization. Additionally, a significant number of antidromic nerve impulses are generated through dorsal root reflexes or local axonal reflexes, which further release these substances peripherally, leading to a neurogenic inflammatory response in the prostate or bladder. This response can exacerbate pain sensitization or may also contribute to bladder dysfunction. Furthermore, NGF influences sensory nerve endings in the prostate or bladder and can be transported from the periphery to DRG neurons. Upon binding to Trk, NGF enhances neuronal excitability, thereby further sensitizing pain. Green arrows represent the effects of different substances on sensory nerves or receptors; brown arrows represent the transport direction of SP, CGRP and NGF; light red dotted arrows represent the direction of nerve impulse transmission. Abbreviations: TNF-α, tumor necrosis factor-alpha; IL-8, interleukin-8; IFN-γ,interferon-gamma; IL-4, interleukin-4; IL-6, interleukin-6; IL-10, interleukin-10; NGF, nerve growth factor; CGRG, calcitonin gene peptide; SP, substance P; DRG, dorsal root ganglion; Trk, tropomyosin receptor kinase; TRPV1, transient receptor potential vanilloid subtype 1; DRG, dorsal root ganglia; C-fiber, capsaicin-sensitive afferent fiber. Image was created with BioRender.com.

In summary, inflammatory changes in pathological prostatitis can trigger the activation of TRPV1 channels in both neural and non-neural tissues, as well as sensitize C-fibers. These sensitized nerve fibers perform a dual role in transmitting pain signals from the periphery to the central nervous system and releasing neuropeptide substances at peripheral nerve endings, potentially intensifying the pain response (Figure 2).

### 4.3 TRPV1 and LUTS caused by CP/CPPS

In addition to pain, patients with chronic prostatitis may also experience LUTS including frequent urination, urgency, and increased nocturia. Clinical data indicates that approximately 50%–60% of patients with chronic prostatitis are affected by LUTS (Nickel, 2012; Magistro and Nickel, 2019). However, The relationship between histological prostatitis and LUTS remains unclear. Some studies suggest a close association between histological prostatitis and LUTS (Chung et al., 2012; Urkmez et al., 2016; Mizuno et al., 2017) while conflicting findings are presented by Kumsar et al. (2016). Discrepancies in these findings may be attributed to variations in patient inclusion criteria. Although the relationship between prostatitis and LUTS has yielded mixed results in clinical studies, this relationship can be elucidated through cross-organ sensitization observed in animal studies.

Cross-organ sensitization is the core link of LUTS. Funahashi et al. (2019) discovered that in rats with right pelvic nerve transection, formalin injection into the right ventral lobe of the prostate did not trigger bladder overactivity. However, injection into the left prostate lobe resulted in more frequent instances of bladder overactivity. Schwartz et al. (2016) discovered in a chemically induced prostatitis model that sensory nerves (afferent nerves) of the prostate and bladder share common afferent signals in the DRG. They also observed that during prostatic inflammation, bladder afferent nerves become sensitized, leading to a significant increase in micturition frequency. The above research data indicate that chronic prostatitis can result in bladder afferent sensitization, ultimately affecting bladder functionality. The DRG innervating both the prostate and bladder serves as the anatomical foundation for the sensitization of the bladder due to prostatitis. Several studies have identified that certain sensory nerves from the prostate and bladder converge in the DRG (Chen et al., 2010; Lee et al., 2016; Schwartz et al., 2016; Funahashi et al., 2019). The DRG serves as the site where the cell bodies of primary afferent neurons from these two organs come together. In cases where the afferent neurons of one organ are triggered by abnormal stimulation or inflammation, neurogenic substances may be generated within the DRG and then transmitted to another organ, leading to neurogenic inflammation in healthy organs and facilitating cross-sensitization between organs (He et al., 2023). As the cell body of various sensory neurons, the DRG also expresses a range of receptors (such as TRPV1 receptors and NGF receptors) and neuropeptide substances (such as SP and CGRP). The interaction of these receptors and neuropeptide substances can lead to a series of neurophysiological activities that play a significant role in the development of LUTS in chronic prostatitis (Haberberger et al., 2019; He et al., 2023).

An increasing number of animal experimental studies demonstrate that chronic prostatitis can result in alterations in bladder function (Chen et al., 2010; Majima et al., 2015; Schwartz et al., 2016; Park et al., 2018; Ni et al., 2019; Aydogdu et al., 2021). One study discovered a correlation between prostatitis-induced bladder overactivity, alterations in TRPV1 in DRG, and heightened expression of neurogenic substances like NGF in the bladder mucosa with the heightened excitability of bladder afferent nerves triggered by prostatitis (Funahashi et al., 2019). The neurogenic substances produced can impact the excitability of bladder afferent neurons (Shimizu et al., 2023; Ni et al., 2022) and the expression of TRPV1 (Shimizu et al., 2018; Igarashi et al., 2021). Additionally, locally overexpressed NGF can promote neurite growth (Ekman et al., 2017), increase the density of bladder afferent neurons (Schnegelsberg et al., 2010), and facilitate the transportation of NGF to the DRG, ultimately up-regulating TRPV1 expression via the NGF-trk pathway (Zhang et al., 2019). When the TRPV1 gene is knocked out, the presence of NGF and trk will not cause bladder overactivity (Frias et al., 2012). This highlights the importance of TRPV1 as a key target in prostatitis-induced bladder overactivity and as a necessary factor for NGF to drive bladder dysfunction (Figure 2).

## 5 TRPV1 channel is a potential therapeutic target for CP/CPPS

Due to TRPV1’s distinctive ability to integrate various nociceptive stimuli, it has been extensively researched and utilized as a target for pain management and other medical conditions (Danigo et al., 2013; Gao et al., 2021; Jaffal, 2023). At varying doses and through different administration methods, TRPV1 can induce three distinct effects on nociceptive nerves: excitation, desensitization, and neurotoxicity (Szallasi and Blumberg, 1999). The natural agonists of the TRPV1 channel include irritating plant products like CAP and RTX, as well as animal toxins and venoms (Fischer et al., 2020). CAP and RTX are the two main vanillic acid compounds. Prolonged exposure to both compounds leads to dephosphorylation and homologous desensitization of the channel in a calcium ion-dependent manner (Aghazadeh Tabrizi et al., 2017), resulting in the “defunctionalization” of sensory neurons and depletion of neuropeptide substances (Iftinca et al., 2021). However, the main difference between the two is that RTX causes a slow and long-lasting depolarization after acting on TRPV1, whereas CAP has the opposite effect (Caterina et al., 1997). This mechanism helps prevent the adverse effects of intense neuronal excitation resulting from CAP’s interaction with TRPV1 (Kissin and Szallasi, 2011).

Preclinical data indicate that TRPV1 channels are significantly involved in the pathogenesis and progression of prostatitis. Modulating TRPV1 channels has the potential to alleviate LUTS and pain associated with prostatitis (Table 1.) (Tang et al., 2007; Park et al., 2018; Roman et al., 2020; Lao et al., 2024; Piao et al., 2024). These studies are currently limited to animal models and have not progressed to clinical research. This could be attributed to a lack of understanding regarding the pathogenesis of prostatitis and the potential side effects of TRPV1. Future research should focus on more extensive zoological and pharmacological investigations to enhance our understanding of prostatitis pathogenesis and to develop new TRPV1 derivatives.

**TABLE 1:** Preclinical data using TRPV1 as a drug target for prostatitis.

Species	Substances to induced prostatitis	Drugs	Drug delivery route	Results	References
Sprague-Dawley male rats	17β-estradiol and dihydrotestosterone	Cannabidiol	Oral administration	Pain relief	Piao et al. (2024)
C57BL/6J (B6) male mices	CFA and Rat male accessory gland extract	Sertraline	intraperitoneal injection	Pain relief; improve depression-like symptoms	Lao et al. (2024)
C57BL/6J (B6) male mices	Rat prostate antigen	Arginine-rich hexapeptide	Via intraurethral instillation	Pain relief	Roman et al. (2020)
Sprague-Dawley male rats	10% buffered formalin	CAP	Subcutaneous injection	Reduced c-fos expression in spinal neurons Reduced bladder overactivity	Park et al. (2018)
Sprague-Dawley male rats	CFA	RTX	Intrathecal administration	Pain relief Reduced incidence of unstable bladder	Tang et al. (2007)

CFA, complete Freund’s adjuvant; CAP, capsaicin; RTX, resiniferatoxin.

In clinical trials, TRPV1 agonists, antagonists, and their derivatives have been extensively utilized across various medical disciplines (Danigo et al., 2013). For instance, 8% capsaicin skin patches have shown efficacy in treating neuropathic pain, liquid capsaicin has been utilized for osteoarthritis management, and Intrathecal administration of RTX has demonstrated effectiveness in alleviating cancer pain (Iftinca et al., 2021). Due to the distribution of TRPV1 channels in various systems of the body (Jaffal, 2023), systemic medication may result in unpredictable adverse reactions. As a result, current medication methods primarily focus on local administration (Iftinca et al., 2021). In most Phase I clinical trials, while TRPV1-related drugs provide pain relief for various patients, they often come with the side effect of elevated body temperature (Bamps et al., 2021). Brown et al. (2017) recently conducted a phase I study to evaluate the safety and pharmacokinetics of oral NEO6860, a mode-selective TRPV1 antagonist, in healthy volunteers. The study found no clinically significant increase in temperature or heat pain threshold/tolerance. Round et al. (2011) investigated the safety of repeated administration of the novel XEN-D0501, a TRPV1 antagonist currently used to treat overactive bladder syndrome, in healthy subjects. They found that the 5 mg dose was safe and well-tolerated, exhibiting good performance with no serious adverse reactions. In comparison to other commonly used TRPV1 antagonists, SB-366791 demonstrates efficient affinity inhibition along with safe and effective selective effects (Gunthorpe et al., 2004). Neuberger et al. (2023) further elucidated the mechanism of interaction between SB-366791 and human TRPV1 following binding and the subsequent conformational changes, thereby advancing the research and application of TRPV1-targeted therapies. Therefore, these clinical studies demonstrate that the systemic application of novel TRPV1 antagonists can significantly mitigate drug-induced adverse reactions, thereby facilitating the advancement of clinical research on TRPV1 medications. Secondly, in terms of topical administration, Turini et al. (2006) found that the topical application of CAP to the perineal skin in patients with CP/CPPS resulted in a significant reduction in the Chronic Prostatitis Symptom Index score and an improvement in pain symptoms. Apostolidis et al. (2006) conducted a follow-up study on patients experiencing refractory urinary urgency and frequency. They found that a single intravesical infusion of RTX could improve the patients’ LUTS, urethral mechanical parameters, and overall quality of life for up to 6 months. Clinical trial studies have demonstrated that intravesical instillation of either RTX or CAP is effective for treating bladder dysfunction resulting from various diseases (Toktanis et al., 2018). Recent studies have demonstrated that several new TRPV1 antagonists have shown promise in the treatment of pain. Notably, Stasi et al. (2022) conducted a study on the topical administration of SAF312, a potent and highly selective non-competitive TRPV1 inhibitor, in healthy subjects. Their findings indicate that this method of administration is both safe and well-tolerated. Furthermore, SAF312 has proven effective in alleviating postoperative pain (Thompson et al., 2023). A randomized, double-blind, controlled study conducted by Segerdahl et al. (2024) demonstrated that the topical application of the novel ACD440 gel, a TRPV1 receptor antagonist, effectively reduced evoked pain responses in healthy volunteers. These clinical studies have demonstrated the role of TRPV1-targeted drugs in the treatment of pain and overactive bladder. Consequently, by integrating the pathogenesis of chronic prostatitis with the function of TRPV1 in the sensitization of internal organs, it is crucial to develop TRPV1-targeted drugs that are safe, efficient, and have minimal side effects for the management of prostatitis in the future.

## 6 Conclusion

This article outlines the mechanism through which prostatitis induces pain and LUTS, with a specific focus on the role and potential therapeutic targets of TRPV1 in prostatitis. Patients with CP/CPPS experience long-lasting pain and increased activity in the bladder, which is caused by cross-sensitization between the prostate and bladder. This cross-sensitization involves the regulation and/or heightened expression of TRPV1 ion channels. Therefore, targeting TRPV1 may be beneficial in controlling CP/CPPS. However, many uncertainties remain regarding the exact mechanism of action and potential therapeutic targets of TRPV1 channels in prostate disease. Further research and clinical trials are needed to improve our understanding and utilization of the potential of TRPV1 channels to treat prostate disease. In summary, the role of TRPV1 channels in prostate diseases holds significant research value and potential therapeutic applications. However, further in-depth research is necessary to elucidate the precise mechanism of TRPV1 channels in prostate diseases and to formulate appropriate treatment strategies.
